# Classifying Suicide Attempts from Suicidal Ideation among Adolescents using Machine Learning

**DOI:** 10.1192/j.eurpsy.2025.1146

**Published:** 2025-08-26

**Authors:** J. Hong, C.-M. Yang

**Affiliations:** 1 Psychiatry, Iksan hospital; 2 Psychiatry, Wonkwang University, Iksan, Korea, Republic Of

## Abstract

**Introduction:**

Suicide is a leading cause of death among adolescents and its prevalence among young people has steadily increased in recent years.

**Objectives:**

This study aimed to identify patterns of risk factors that differentiate adolescents who experienced suicidal thoughts from those who attempted suicide using six different machine learning (ML) algorithms for Korean adolescents using data from online surveys.

**Methods:**

Data were extracted from the 2011−2018 Korea Youth Risk Behavior Survey (KYRBS), conducted annually since 2005 by the Korean Ministry of Education, Ministry of Health and Welfare, and Korean Disease Control and Prevention Agency. The pipeline was solely generated from classic ML (CML) methods, namely logistic regression (LR), random forest (RF), artificial neural networks (ANN), support vector machines (SVM), and extreme gradient boosting (XGB).

**Results:**

Among the 69,840 adolescents included in the analysis, 13,288 cases (19.0%) were identified as having made a suicide attempt. Prediction models using seven relevant features calculated by Boruta algorithm was developed and five features (drug experience, current smoking, grade, current alcohol drinking and sadness or hopelessness) were identified as the most important predictors. The performance of the six ML models on the balanced testing dataset was good, with area under the receiver operating characteristic curve (AUROC) and area under the precision−recall curve (AUPRC) ranging from 0.66 to 0.73.

**Image 1:**

**Figure:**
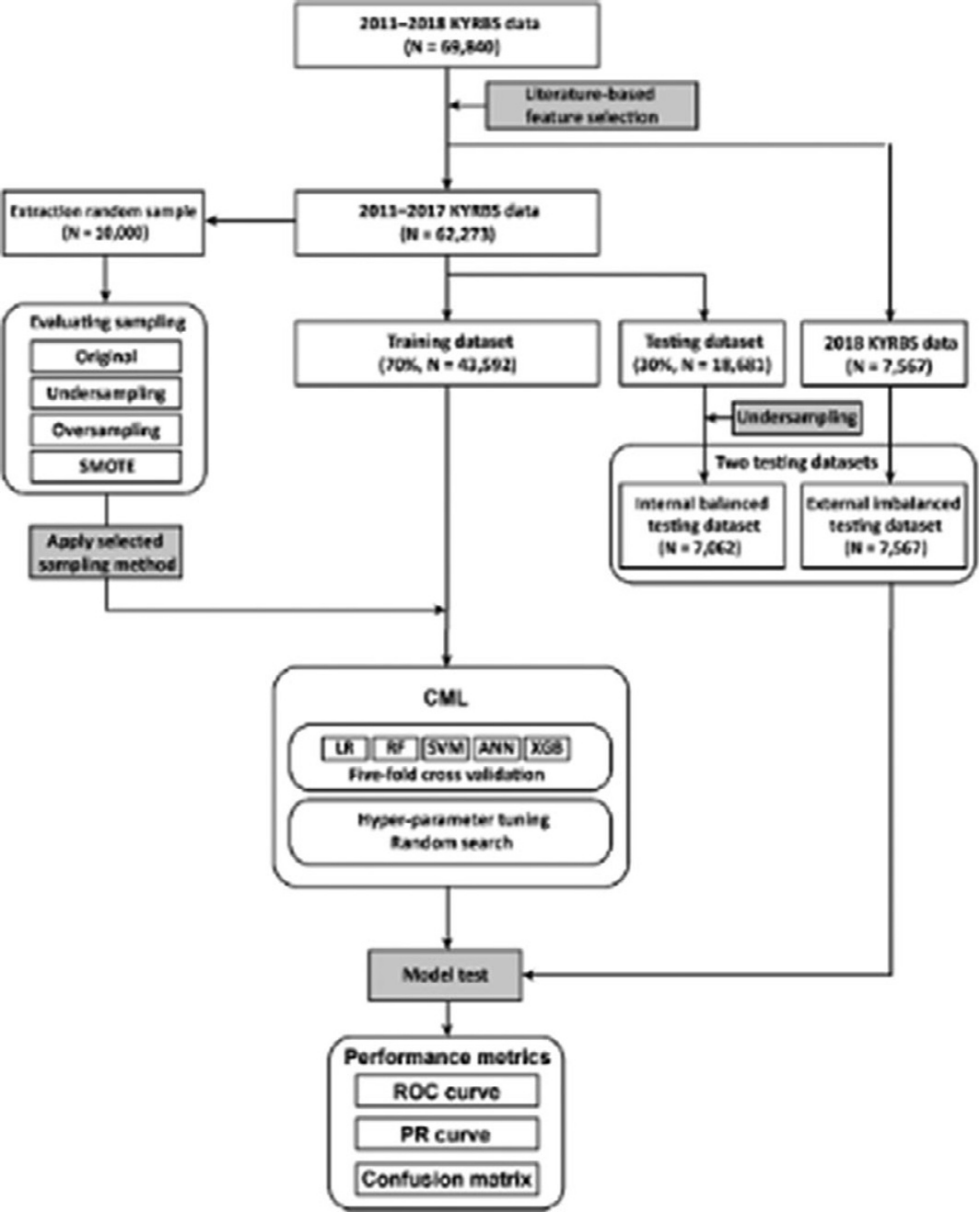

**Image 2:**

**Figure:**
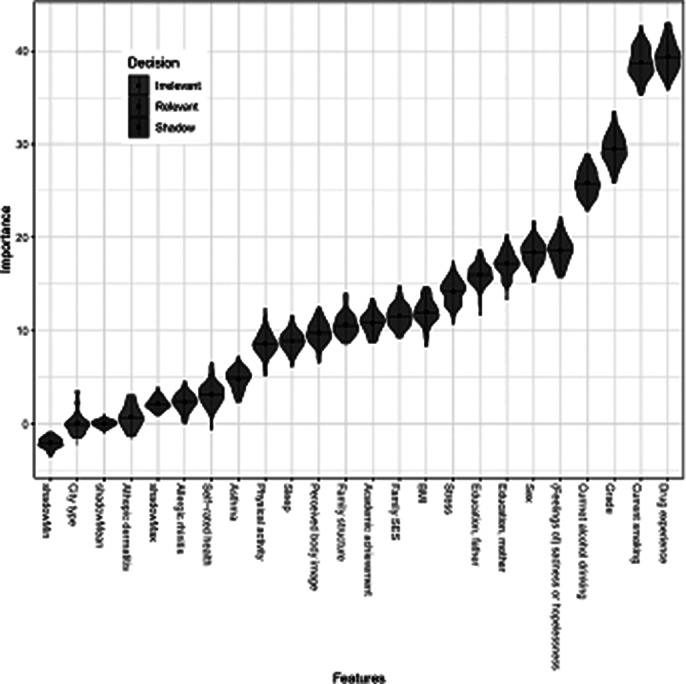

**Image 3:**

**Figure:**
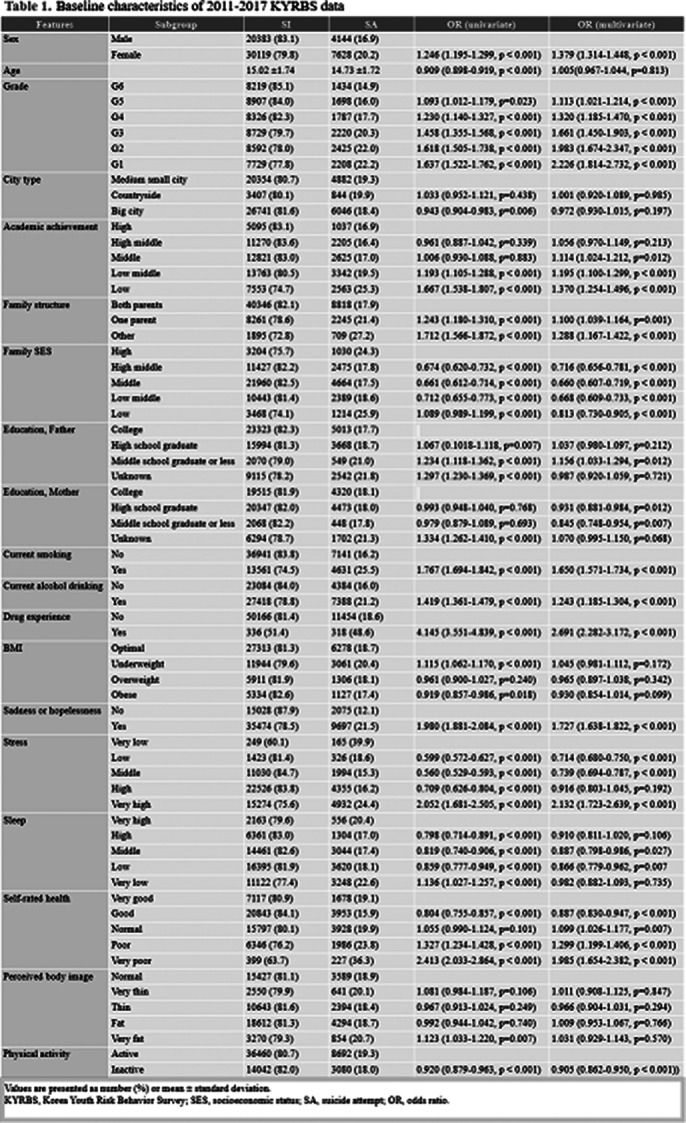

**Conclusions:**

The developed and validated SA prediction models can be applied to detect high risks of SA. This approach could facilitate early intervention in the suicide crisis and may ultimately contribute to suicide prevention for adolescents.

**Disclosure of Interest:**

None Declared

